# Evidence-based health messages increase intention to cope with loneliness in Germany: a randomized controlled online trial

**DOI:** 10.1038/s41746-024-01096-7

**Published:** 2024-04-29

**Authors:** Shuyan Liu, Matthias Haucke, Luisa Wegner, Jennifer Gates, Till Bärnighausen, Maya Adam

**Affiliations:** 1 Department of Psychiatry and Psychotherapy (Campus Charité Mitte), Charité—Universitätsmedizin Berlin, Berlin, Germany; 2 German Center for Mental Health (DZPG), Berlin and Heidelberg, Germany; 3 Department of Education and Psychology, Freie Universität Berlin, Berlin, Germany; 4 Columbia University Mailman School of Public Health, New York, NY USA; 5 Icahn School of Medicine at Mt. Sinai, New York, NY USA; 6 Heidelberg Institute of Global Health (HIGH), Heidelberg University, Heidelberg, Germany; 7 Africa Health Research Institute, Durban, South Africa; 8 Harvard Center for Population and Development Studies, Cambridge, MA USA; 9 Department of Pediatrics, Stanford University School of Medicine, Stanford, CA USA; 10 Center for Digital Health, Department of Medicine, Stanford University School of Medicine, Stanford, CA USA

**Keywords:** Quality of life, Education

## Abstract

Loneliness poses a formidable global health challenge in our volatile, post-pandemic world. Prior studies have identified promising interventions to alleviate loneliness, however, little is known about their effectiveness. This study measured the effectiveness of educational entertainment (“edutainment”) and/or evidence-based, written health messages in alleviating loneliness and increasing intention to cope with loneliness. We recruited 1639 German participants, aged 18 years or older. We compared three intervention groups who received: (A) edutainment and written health messages, (B) only edutainment, or (C) only written health messages, against (D) a control group that received nothing. The primary outcomes were loneliness and intention to cope with loneliness. Participants were also invited to leave comments about the interventions or about their perception or experiences with loneliness. We found a small (*d* = 0.254) but significant effect of the written messages on increased intention to cope with loneliness (*b* = 1.78, *t*(1602) = 2.91, *P* = 0.004), while a combination of edutainment and written messages significantly decreased loneliness scores (*b* = −0.25, *t*(1602) = −2.06, *P* = 0.04) when compared with the control, even after adjusting for covariables including baseline values, self-esteem, self-efficacy, and hope. We also observed significantly higher self-esteem scores after exposure to a combination of edutainment and written messages (*b* = 0.821, *t*(1609) = 1.76, one-tailed *P* = 0.039) and significantly higher hope scores after exposure to edutainment-only (*b* = 0.986, *t*(1609) = 1.85, one-tailed *P* = 0.032) when compared with the control group. Our study highlights the benefits of using written messages for increasing intention to cope with loneliness and a combination of edutainment and written messages for easing loneliness. Even in small “doses” (less than 6 min of exposure), edutainment can nurture hope, and edutainment combined with written messages can boost self-esteem.

## Introduction

Loneliness is a common problem worldwide^1,2^. One in four EU citizens reported feeling lonely frequently in 2020^3^. The risk factors for loneliness include gender, limited use of coping strategies, and low education, self-esteem, and self-efficacy^4–6^. Loneliness is a documented contributor to psychopathological symptoms^7^, yet there is a lack of research exploring and evaluating the effectiveness of innovative, scalable interventions designed to reduce loneliness^8,9^.

Prior research has explored various face-to-face interventions aimed at reducing loneliness. These include social skills training, building social support and social contact, and providing psychoeducation and cognitive behavioural training^10,11^. While effective, such face-to-face trainings can be hard to scale across large populations, limiting access and ability to impact large numbers of individuals. Online delivery can be used to increase access and a recent study found that a 10-week internet-based study effectively alleviated loneliness through self-help interventions, with human guidance and automated messages^8^. However, even online, accessible interventions may demand significant time investments (i.e., weeks to months) which limit their scalability to broad public audiences. An innovative and efficient method to educate individuals about loneliness and other health issues exists through entertainment-education, also known as “edutainment”^12,13^. A specialised application of entertainment-education, *short, animated storytelling* uses short-form, often wordless animations to broadly disseminate health messages globally via social media and the internet. This short-form animated storytelling emerged during the COVID-19 pandemic as a way of rapidly spreading critical health messages globally, while surpassing culture, language and literacy barriers^14,15^.

Edutainment is designed to integrate health-promoting, educational, prosocial and destigmatizing messages in entertainment media with the intent to influence awareness, knowledge, attitudes, and even behaviour of the viewers^16,17^. This approach is grounded in the social cognitive learning and behaviour change theory^16,18^ which posits that our values and behaviours can be affected by observing, modelling, and imitating others’ behaviour, attitudes, or emotional expressions. In fact, edutainment has been used to successfully address mental health challenges such as reducing stigma and improving mental health literacy^19–21^. The foundations of edutainment and other health outreach strategies rely on robust evidence in order to effectively promote public health^22^. Health behaviour change interventions are often criticised for lacking this solid research evidence base^23^. Evidence-based health messages are needed to raise awareness, combat misinformation and reinforce behaviour change^24^.

Our behaviours are determined, in part, by our intentions to perform the behaviour^25,26^. Intentions, in turn, are influenced by attitudes, subjective norms, and perceived behavioural control^25,26^. Prior reviews indicate that behavioural intentions can explain between 18 and 23% of the variance in individual health behaviours^27–29^. Medium-to-large changes in behavioural intentions lead to small-to-medium changes in behaviour^29,30^. In the context of loneliness, edutainment and evidence-based health messages may boost our intention to cope with loneliness by changing our negative evaluation of loneliness—for example, an individual reframing loneliness as an opportunity for reflection. These health messages can also shape perceptions of social pressure from others and build confidence in overcoming loneliness, such as increasing one’s belief that they possess the strength and resources to overcome loneliness. Our pilot study showed that edutainment interventions have the potential to increase individuals’ intention to cope with loneliness^31^. However, we could not significantly quantify its effectiveness due to a limited sample size (*N* = 252). There is a pressing need to measure the effectiveness of innovative health edutainment interventions in order for them to be used to promote public health globally at scale^8,9,32^.

Encouraged by the results of our pilot study^31^, This study aims to establish the effectiveness of edutainment and/or evidence-based, written messages in alleviating feelings of loneliness and increasing intention to cope with loneliness. We hypothesise that a combination of edutainment and written messages will enhance intention to cope with loneliness and reduce loneliness. This combined approach could also boost self-esteem and self-efficacy, while nurturing hope.

## Results

### Participants characteristics

A total of 1689 participants accessed our study and 1639 of them (802 females; age range: 18–71, mean = 28.28, SD = 8.59) completed our study. Sociodemographic characteristics in each arm are shown in Table 1. Approximately 94% of participants were vaccinated twice or more against COVID-19 in each trial arm, reflecting similar attitudes toward COVID-19 vaccination across four trial arms. The mean ULS-8 loneliness scores before and after intervention were 16.93 and 16.68 (range from 8 to 31), respectively. The mean intention to cope with loneliness score before and after intervention were 64.36 and 65.29 (range from 13 to 99), respectively.

**Table 1:** Sociodemographic characteristics of participants (*N* = 1613)

Variable	Edutainment and message (*N* = 407)	Edutainment (*N* = 407)	Message (*N* = 402)	Control (*N* = 397)
Age (years), mean (SD)	28.6 (9.01)	28.5 (8.67)	27.9 (8.78)	28.3 (7.88)
Gender, *n* (%)				
Woman	194 (48%)	199 (49%)	191 (48%)	207 (52%)
Man	206 (50%)	203 (50%)	201 (50%)	182 (46%)
Other	7 (2%)	5 (1%)	10 (2%)	8 (2%)
Education years, mean (SD)	15.8 (3.33)	16.0 (3.82)	16.0 (3.34)	16.4 (3.13)
Vaccinated, *n* (%)				
No	16 (4%)	16 (4%)	12 (3%)	16 (4%)
Once	10 (2%)	6 (1%)	5 (1%)	2 (1%)
Twice	89 (22%)	88 (22%)	88 (22%)	75 (19%)
>Twice	288 (71%)	296 (73%)	294 (73%)	298 (75%)
Not to say	4 (1%)	1 (0%)	3 (1%)	6 (1%)
ULS-8 loneliness score before intervention, mean (SD)	16.8 (5.02)	16.7 (4.71)	17.4 (5.03)	16.8 (4.88)
ULS-8 loneliness score after intervention, mean (SD)	16.5 (5.16)	16.4 (5.01)	17.1 (5.05)	16.7 (4.92)
Intention to cope with loneliness score before intervention, mean (SD)	64.4 (11.1)	64.6 (11.4)	64.1 (11.0)	64.4 (11.4)
Intention to cope with loneliness score after intervention, mean (SD)	65.7 (14.4)	65.6 (14.4)	65.7 (13.1)	64.0 (12.6)
Hope score (AHS) after intervention, mean (SD)	62.3 (7.22)	62.7 (7.78)	62.4 (7.54)	61.7 (7.72)
Self-esteem score (RSE) after intervention, mean (SD)	28.8 (6.17)	28.1 (6.88)	27.7 (6.57)	28.0 (6.84)
Self-efficacy score (GSES) after intervention, mean (SD)	28.1 (5.17)	28.0 (5.27)	27.5 (5.30)	27.7 (5.30)

*SD* standard deviation, *ULS-8* short-form UCLA Loneliness Scale, *AHS* Adult Hope Scale, *RSE* Rosenberg Self-Esteem Scale, *GSES* General Self-Efficacy Scale.

### Effectiveness of edutainment and written messages on loneliness and intention to cope with loneliness

**Fig. 1:**
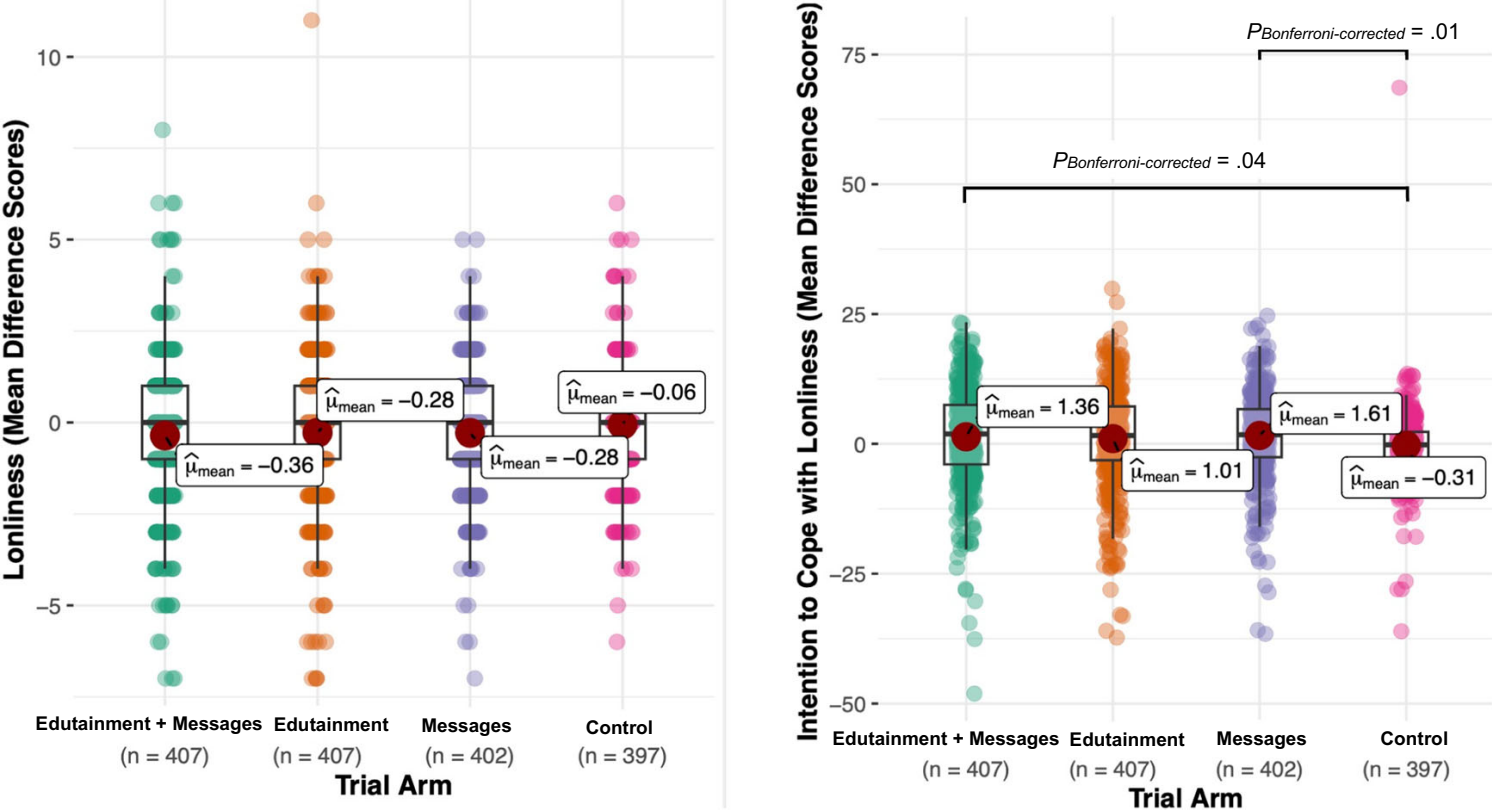
Primary outcomes before and after intervention. Mean difference between loneliness and intention to cope with loneliness scores before and after intervention across the four trial arms.

We tested the overall effectiveness of each intervention independent of the specific type of intervention by using the independent-samples *t* test. We found an overall effectiveness across the interventions (on loneliness and intention to cope with loneliness scores) that was independent of the specific type of intervention. Specifically, there was a significant mean reduction in loneliness post-intervention (mean = 16.67, SD = 5.08) compared to pre-intervention (mean = 16.97, SD = 4.92), *t* = −5.69, *df* = 1215, *P* < 0.001. As shown in Fig. 1, the mean difference in the loneliness score was −0.30 (95% CI: −0.41 to −0.20). There was also a significant mean increase in the intention to cope with loneliness post-intervention, mean = 65.69, SD = 13.97, compared with pre-intervention, mean = 64.37, SD = 11.17 (*t* = −5.69, *df* = 1215, *P* < 0.001). The mean difference in the intention to cope with loneliness score was 1.33 (95% CI: 0.80–1.85). Figure 1 shows the mean difference between loneliness and intention to cope with loneliness, before and after intervention across the four trial arms.

To test the effects of each specific type of intervention, we conducted one multiple regression analysis on loneliness and another on intention to cope with loneliness. All generalised variance-inflation factors (GVIF) were less than 2.60. As displayed in Tables 2 and 3, our multiple regression results showed that a combination of edutainment and written messages significantly decreased loneliness scores when compared with the control condition, even after adjusting for covariables including baseline value before intervention (*b* = −0.25, *t*(1602) = −2.06, *P* = 0.04). Neither the edutainment-only nor the written-messages-only interventions demonstrated significant effects (both *P* values > 0.08). We did observe significant increases in intention to cope with loneliness after exposure to both the combined edutainment + written messages (*b* = 1.48, *t*(1602) = 2.41, *P* = 0.02) as well as the written messages only intervention (*b* = 1.78, *t*(1602) = 2.91, *P* = 0.004), while the edutainment-only intervention did not significantly increase intention to cope (*P* = 0.06). Both baseline values of loneliness (*b* = 0.91, *t*(1602) = 78.94, *P* < 0.0001) and intention to cope with loneliness (*b* = 0.91, *t*(1602) = 44.73, *P* < 0.0001) significantly predicted their post-intervention scores. Interestingly, we found that high self-esteem was linked to lower loneliness after intervention (*b* = −0.046, *t*(1602) = −4.40, *P* < 0.0001) but similar associations were not observed with self-efficacy and hope. On the other hand, higher hope scores appeared to be significantly associated with increased intention to cope with loneliness (*b* = 0.149, *t*(1602) = 4.13, *P* < 0.0001). In Table 2 we present the results our multiple regression analysis of the sum loneliness scores, after intervention.

**Table 2:** Multiple regression results of the sum loneliness score after intervention as an outcome

Variable	*b*	SE *b*	*T* value	*P* value
Edutainment and message versus control	−0.254	0.123	−2.062	0.04
Edutainment versus control	−0.215	0.123	−1.744	0.08
Message versus control	−0.184	0.124	−1.488	0.13
Covariates				
Baseline loneliness scores before intervention	0.910	0.012	78.940	<0.0001
Hope	−0.002	0.007	−0.345	0.73
Self-esteem	−0.046	0.010	−4.397	<0.0001
Self-efficacy	−0.020	0.013	−1.503	0.13
Age	0.019	0.006	3.389	<0.0001
Gender	0.189	0.082	2.301	0.02
Years of education	0.005	0.013	0.361	0.72
Degrees of freedom: 1602

**Table 3:** Multiple regression results of the mean intention to cope with loneliness score after an intervention as an outcome

Variable	*b*	SE *b*	*T* value	*P* value
Edutainment and message versus control	1.475	0.612	2.410	0.02
Edutainment versus control	1.147	0.611	1.876	0.06
Message versus control	1.784	0.613	2.910	0.004
Covariates				
Baseline intention to cope with loneliness scores before intervention	0.906	0.020	44.727	<0.0001
Hope	0.149	0.036	4.127	<0.0001
Self-esteem	0.053	0.047	1.133	0.26
Self-efficacy	−0.046	0.065	−0.696	0.49
Age	0.034	0.027	1.264	0.21
Gender	−0.736	0.410	−1.793	0.07
Years of education	−0.165	0.067	−2.452	0.01
Degrees of freedom: 1602				

In Table 3 we present the results our multiple regression analysis of the mean intention to cope with loneliness scores, after intervention.

To calculate effect sizes, we conducted a pairwise comparison of loneliness and intention to cope with loneliness scores before and after intervention, as shown in Tables 4 and 5. According to Cohen’s classification of effect sizes of small (*d* = 0.2), medium (*d* = 0.5), and large (*d* = 0.8)^33^, we found a small but significant increase in intention to cope with loneliness after exposure to the written messages only (*d* = 0.254).

**Table 4:** Effect sizes (Cohen *d*) for the difference in the sum loneliness scores for each arm

Trial arm	*T* test (*df*)	Bonferroni adjusted *P* values	Effect size (Cohen *d*)
Edutainment and message versus control	−2.43 (742)	0.11	−0.171
Edutainment versus control	−1.76 (735)	0.50	−0.124
Message versus control	−2.07 (790)	0.46	−0.147
Edutainment and message versus Edutainment	−0.56 (812)	>0.99	−0.039
Edutainment and message versus message	−0.58 (777)	>0.99	−0.040
Edutainment versus message	0.05 (771)	>0.99	0.003

**Table 5:** Effect sizes (Cohen *d*) for the difference in the mean intention to cope with loneliness scores for each arm

Trial arm	*T* test (*df*)	Bonferroni adjusted *P* values	Effect size (Cohen *d*)
Edutainment and message versus control	2.87 (715)	0.04	0.202
Edutainment versus control	2.23 (710)	0.20	0.157
Message versus control	3.59 (757)	0.01	0.254
Edutainment and message versus Edutainment	0.52 (812)	>0.99	0.036
Edutainment and message versus message	−0.39 (794)	>0.99	−0.027
Edutainment versus message	−0.94 (791)	>0.99	−0.066

### Effectiveness of edutainment and written messages on self-esteem, self-efficacy, and hope

**Fig. 2:**
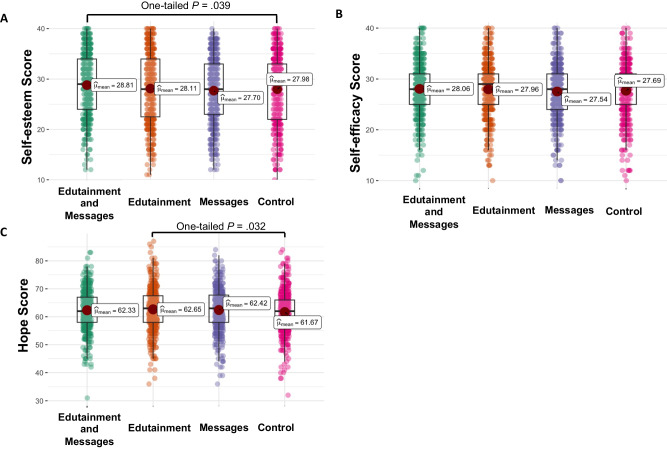
Secondary outcomes after intervention. The differential effects of a combination of edutainment and written messages, edutainment-only, and written messages only against a control condition on scores of self-esteem (**A**), self-efficacy (**B**), and hope (**C**). Significant at *p* (one-tailed test).

To analyse secondary outcomes, we reported one-tailed *P* values as we hypothesized that edutainment and written messages will enhance self-esteem, self-efficacy, and hope. Our linear regression results revealed significantly higher self-esteem scores after exposure to a combination of edutainment and written messages (*b* = 0.821, *t*(1609) = 1.76, one-tailed *P* = 0.039) and significantly higher hope scores after exposure to edutainment-only (*b* = 0.986, *t*(1609) = 1.85, one-tailed *P* = 0.032) when compared with the control, as shown in Fig. 2.

### Emotional responses to edutainment and written messages

**Fig. 3:**
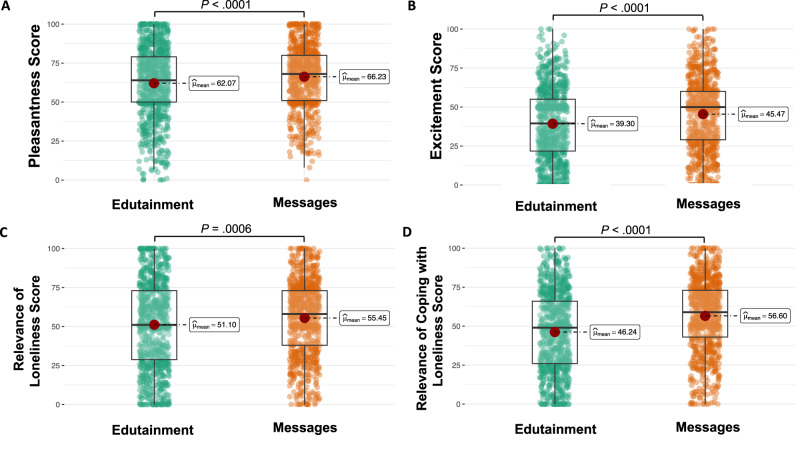
Responses to edutainment and written messages. Emotional responses (pleasantness (**A**) and excitement (**B**)) and the relevance of loneliness (**C**) and coping with loneliness (**D**) to edutainment and written messages.

Finally, we evaluated participants’ emotional responses to the edutainment and written messages. Our results showed significantly higher valence/pleasantness (*t* (1644) = −3.95, *P* < 0.0001), arousal/excitement (*t* (1644) = −5.49, *P* < 0.0001), relevance of loneliness (*t* (1644) = −3.43, *P* = 0.0006), and coping with loneliness (*t* (1644) = −8.65, *P* < 0.0001) in response to written messages when compared with edutainment-only, as shown in Fig. 3.

### Summary of open-ended feedback

While we did not conduct a formal thematic analysis of the open-ended feedback offered by participants, we summarise the main feedback we received from participants here:

In the open-ended feedback box, at the end of the study, some participants noted that the written messages elicited a feeling of solidarity in the shared experience of loneliness. One participant wrote: “written messages reflected that we are not alone in our loneliness, and we are all in the same boat when it comes to loneliness. So, we do not have to feel so lonely, and we can decide to do something about loneliness”. Some participants also wrote that they intended to reframe lonely times as times for reflection. After reading the written messages, they felt they had the strength and resources necessary to overcome their loneliness. They felt more willing to engage in activities they love (e.g., taking up a sport and a hobby) and turn to their friends for companionship and support.

Regarding the edutainment messages, some participants commented in the last open-ended question that the edutainment intervention was an emotionally touching video. They also mentioned that despite feeling isolated, the edutainment video reminded them that they are not alone and encouraged help-seeking. Moreover, participants wrote that a combination of edutainment and written messages could help people become more self-aware and know themselves better. For instance, one participant (ID: 1400) left a comment that “a combined approach can help us better understand loneliness and how it feels like, and it also highlights the importance of cohesion and solidarity”.

Some participants also offered valuable, constructive feedback about the interventions. For example, several participants suggested keeping the edutainment videos short to optimise engagement. Others wanted to know where they could listen to inspiring stories of people who overcame loneliness. These comments support the short, animated storytelling approach used in our edutainment interventions. Participants also shared dissemination ideas, suggesting that evidence-based health messages should be placed on streets and played in television news generally.

## Discussion

Our study investigated the effectiveness of edutainment and/or written messages on loneliness and intentions to cope with loneliness. We found a significant decrease in loneliness scores after exposure to a combination of edutainment and written messages, after controlling for baseline values. We also observed a small but significant effect of the written messages alone on intention to cope with loneliness. We found that exposure to edutainment can boost hope, while a combination of edutainment and written messages can boost self-esteem.

A combination of edutainment and written messages can be a promising approach to easing feelings of loneliness, and written messages alone can enhance one’s intentions to cope with loneliness. The combined approach, edutainment and written messages, significantly reduced feelings of loneliness, although we saw no evidence that this combined approach enhanced intention to cope with loneliness. The differential effects of edutainment on loneliness and intention to cope with loneliness highlight the gap between behaviours and behavioural intentions^29^. Our edutainment video featured an emotion-driven, story-based portrayal of common loneliness experiences and encouraged help-seeking behaviour. After watching it, participants felt less lonely, but this improvement did not appear to translate into conscious plans to cope with those feelings.

Evidence-based health messages, alone, however, did make participants feel like taking action to tackle their feelings of loneliness. One potential explanation of this finding is that participants who felt less lonely after a single exposure to an edutainment video may be less motivated in the moment to express intentions to cope with similar feelings in the future. The tendency to withdraw and believe that one’s struggles are unique is associated with exacerbated suffering during challenging emotional states, such as loneliness^34^. Since the written messages described loneliness as a common experience, this information may have helped participants understand that, even if they were feeling lonely, they were not alone in their experience. The written messages also may have introduced the idea that loneliness can be temporary, and people can reframe the feeling, possibly leaving participants feeling better prepared to cope with loneliness in the future. This explanation is underscored by feedback from the open-ended comments, in which participants described feeling a) comforted by the fact that loneliness is relatively common and b) empowered to reframe periods of loneliness as time for reflection and other activities they perceived as meaningful. These results may reflect changes in attitude, subjective norm, and perceived behavioural control after reading written messages^25,26^.

In this study, we hypothesised that a combination of edutainment and written messages would also boost self-esteem, self-efficacy, and hope. Our hypothesis was partially validated, in that the combined approach enhanced self-esteem, while the edutainment-only approach boosted hope. We observed no significant changes in self-efficacy.

In line with previous studies, our study underscores that edutainment can be an effective method for scaling public health messages, boosting engagement and conveying positive emotions^14,15,35–37^. According to the emotional flow hypothesis^38^, edutainment can catalyse shifts in the emotional state^39^, from fear of being lonely to hope for belonging^40^. One of the key new takeaways from this study, emerges from our observation that combining written and edutainment messages may be particularly effective for engaging audiences. One participant (ID: 1184) suggested that a mixture of emotional content and scientific facts may be the most effective as emotions are the best way to reach people and scientific facts may be beneficial for long-term education. Another participant (ID: 538) added that a combined approach could reach broad populations regardless of whether people prefer creative, entertaining videos or scientific facts.

There are some limitations to the study presented here. Firstly, online populations may not be representative of the entire population due to self-selection and the digital divide. We also only evaluated the immediate short-term effects of the intervention with no follow-up. Thus, we were not able to measure the long-term treatment effects. Longitudinal studies are needed to determine whether the observed effects are sustained over time^9^. In addition, we relied on participants’ self-report which may be subject to social desirability bias, although we would expect this bias to be equal across groups and therefore, we interpret the observed differences between our study groups to be true indications of an intervention effect. Further objective measures (e.g., pupil dilation, skin conductance, and heart rate) could serve as powerful complements to self-report data. Future studies might also test different types of messaging strategies (e.g., rational and emotional framing, attitudinal and normative messages) as these may have differential effects on easing loneliness. Despite these limitations, our study underscores the potential for innovative, scalable solutions, combining short, animated storytelling with evidence-based, written health messages, to alleviate loneliness in the public. This is especially important at a turbulent time when we face widespread loneliness amidst a global mental health crisis.

Our study could help researchers and practitioners, in the field of public health communication, to better understand how visual storytelling might be used as a powerful tool for addressing loneliness, by increasing the accessibility of evidence-based health messages. Our findings add to a growing body of research on “short, animated storytelling” (SAS)—an innovative approach to rapid, global health communication. Such interventions can be cost-effectively disseminated, via social media, by health service providers, public health agencies, schools, and universities. The SAS approach is gaining credibility and could be used for a myriad of other health promotion topics. Additional studies are needed to measure the effect of this approach on other outcomes related to mental and physical health. Because our health is inextricably linked to our human stories, the appeal of these stories can be used to engage the public and transmit messages that improve human health at all levels.

## Methods

### Participants and Procedure

**Fig. 4:**
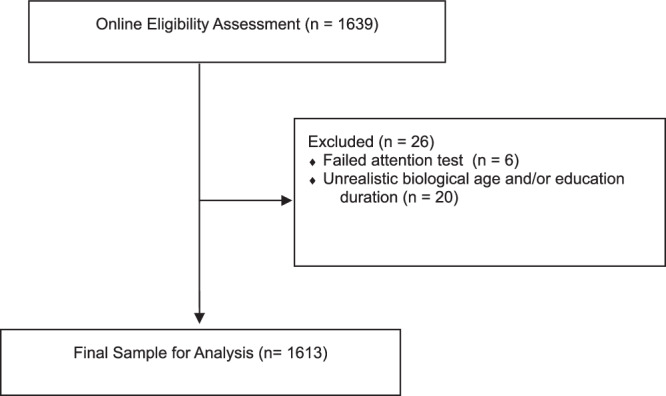
Recruitment flow. Eligibility, exclusions and final sample for analysis.

We recruited participants through an online academic research platform, Prolific (https://www.prolific.co). Eligible participants were: (1) age 18 years or older, (2) fluent in German, and (3) residents of Germany at the time of the trial. All participants provided informed consent online prior to enrolment. The randomised controlled online trial was conducted in Germany from July 8th, 2022 to December 10th, 2022, hosted on the Unipark online research and survey platform^41^. Each participant received GBP £2.8 (EUR €3.3) compensation for participation in the 20-min experiment. The entire experiment took place online. The recruitment is shown in Fig. 4. We excluded 6 participants who had more than 50% wrong answers in the attention check questions and 20 participants whose years of education were more than their biological age or less than 9 years (i.e., years of compulsory education in Germany). Thereby, we analysed data of 1613 participants.

### Ethical considerations

The study was performed in accordance with the ethical standards laid down in the 1964 Declaration of Helsinki and was approved by the Ethics Committee of Charité—Universitätsmedizin Berlin (ethics registration number: EA4/022/22). We followed the CONSORT guidelines^42^, and Good Clinical Practice guidelines^43^ and registered our study on the German Clinical Trials Register^44^ on April 7th, 2022 (trial registration number #DRKS00028748). The study protocol was published as a pilot study^31^.

### Trial design and study intervention

**Fig. 5:**
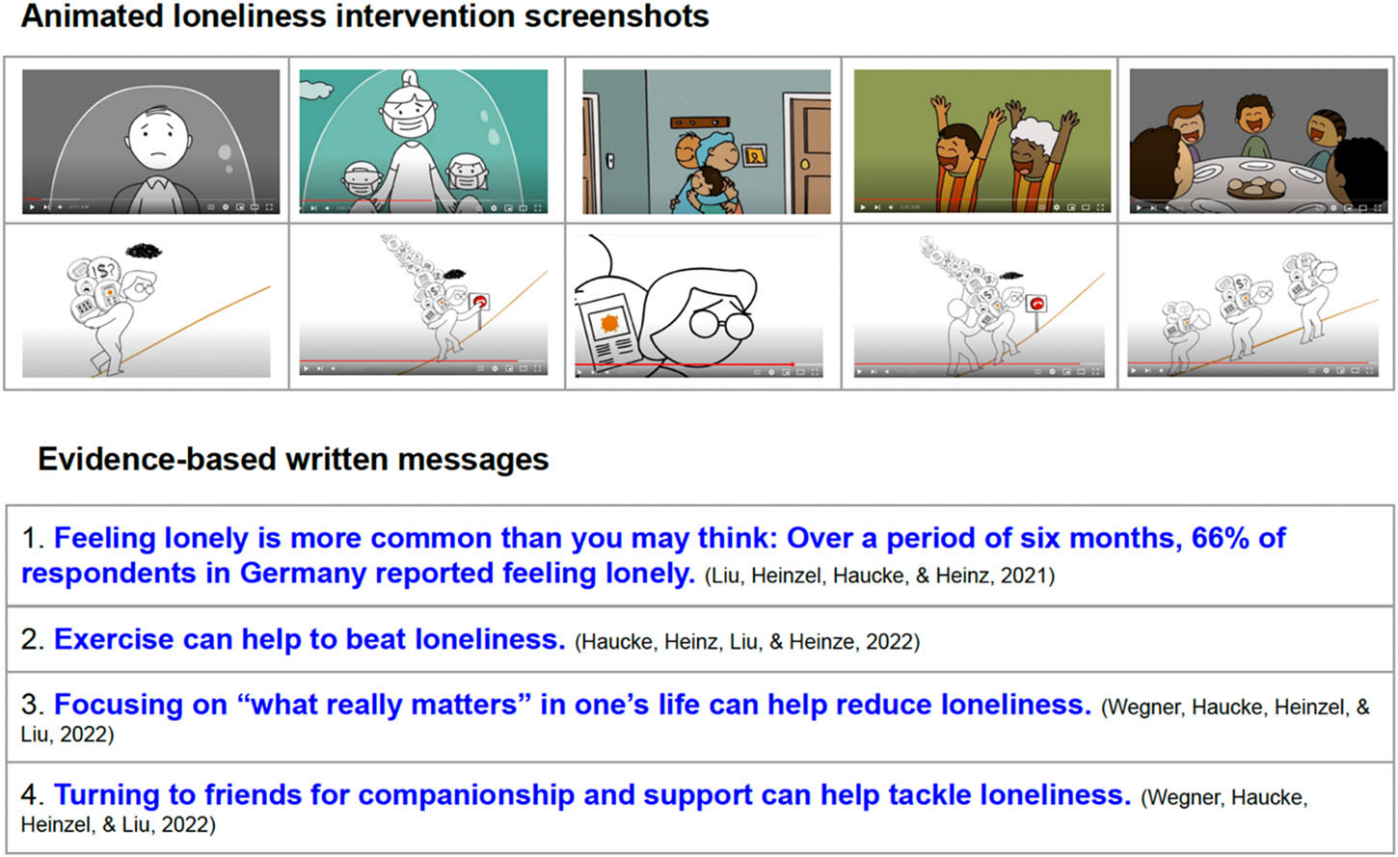
Intervention screenshots. Animated EE video and evidence-based written messages.

We used a factorial trial design (4 conditions) and implemented the interventions (A, B, and C) and the control condition (D) to assess the effectiveness of edutainment and written messages on alleviating feelings of loneliness and increasing intention to cope with loneliness. Each participant was exposed to the intervention only once and was randomised 1:1:1:1 to those four conditions, using the randomisation feature of the online Unipark platform on which our trial takes place. In Arm A, participants watched a 4:39-min animated video and then read the written messages (85 words in German) shown in Fig. 5. Importantly, the animated video did not contain any spoken or written language. Written messages did not contain any sound or animation and conveyed four scientific facts about loneliness, in German. Participants in Arm B watched the animated video only. Participants in Arm C read the written messages only. In the control condition, participants did not receive any intervention. Both the study investigators and analysts were blinded to the study condition assignment.

The wordless animated video was developed by our co-author at Stanford University School of Medicine and can be viewed on YouTube (https://youtu.be/JlzE4rq45u0)^45^. Screenshots from the animated intervention are shown in Fig. 5. The video conveyed a pressing sense of social isolation and loneliness, reflected the potential downsides of remaining isolated when re-engaging with others is an option, and promoted hope, self-efficacy, and help-seeking behaviours.

The written messages convey four key research results of our loneliness studies in Germany (also shown in Fig. 5): (1) Feeling lonely is more common than you may think: Over a period of six months, 66% of respondents in Germany reported feeling lonely^46^, (2) Exercise can help to beat loneliness^47^, (3) Focusing on “what really matters” in one’s life can help reduce loneliness^48^ and (4) Turning to friends for companionship and support can help tackle loneliness^48^.

### Outcomes

The primary outcomes were measured before the intervention introduction and after the intervention completion without interval of time. And the secondary outcomes were only measured after the intervention, utilising Unipark. The primary outcome measures used to quantify loneliness were the aggregate score of the short-form UCLA Loneliness Scale (ULS-8)^49^ and the mean score of the 10 items from the Coping with Loneliness Questionnaire^50^. Both scales have been validated and used in our previous studies^31,46,51^. The 8-item ULS-8 was rated on a four-point Likert response scale, with a total score ranging from 8 to 32. Higher scores indicate a higher level of loneliness. The intention to cope with loneliness was rated on a visual analogue scale (range from 0 to 100). Higher scores indicate higher intention to cope with loneliness.

Our secondary outcomes, self-esteem, self-efficacy, and hope, were measured by using the 10-item Rosenberg Self-Esteem Scale (RSE)^52^, the 10-item General Self-efficacy Scale (GSES)^53^, and the 12-item Adult Hope Scale (AHS)^54^, respectively. These standard scales have been validated in a German context^31,48,55–58^. RSE and GSES were rated on a 4-point Likert response scale, with a total score ranging from 10 to 40. Higher scores indicate higher self-esteem and self-efficacy. AHS was rated on an 8-point Likert response scale, with a total score ranging from 12 to 96. Higher scores indicate higher hope.

Moreover, to ensure participants were paying attention to each intervention, we included 8 attention check items in Arm A, 4 items in Arm B, and 4 items in Arm C, to ensure that participants had thoroughly watched the video and read the messages. Participants who had more than 50% wrong answers on these attention-check questions were removed from the analysis.

At the end of the study, we assessed participants’ emotional responses to the stimuli, vaccination status, as well as sociodemographic characteristics including age, gender, and years of education. We used the horizontal visual analogue scale, ranging from 0 (not at all) to 100 (very much), to ask participants to rate valence/pleasantness, arousal/excitement^59^, and loneliness/coping relevance for both edutainment and written messages. Beyond quantitative assessments, we also included an open-ended question to allow participants to share feedback about their experiences of watching the edutainment video and reading the written messages, or other comments related to loneliness and coping with loneliness.

### Sample size and power considerations

We estimated the sample size based on the outcomes of our pilot study^31^. In that study, there were no statistically significant difference in loneliness scores between the four conditions (Control, Edutainment, Message, Edutainment + Message) on loneliness (*F*(3, 1) = 0.92, *p* value = 0.43, partial eta squared (*η*^2^_*p*_) = 0.01. We utilised the estimated effect size to determine the necessary sample size for our current study. By converting the partial eta squared ed (*η*²_*p*_) value to Cohen’s f using the formula: f = sqr (eta^2 / (1 - eta^2), we obtained Cohen’s f = 0.11 and uncovered a small effect size^33^. In addition, we calculated the correlation between pre- and post-scores of loneliness, yielding a correlation coefficient (*r*) of 0.93. Using the G*power software^60^, we conducted a sample size estimation, indicating a requirement of 1492 participants to achieve a power of 0.95. Considering a drop-out rate of 10%, we determined the necessary sample size to be 1641 participants. For more details, please refer to the supplementary information and Supplementary Fig. 1.

### Data analysis

We used the R version 4.1.0 Statistical Software (R Foundation for Statistical Computing) to analyse our data. To meet the assumption of having no multicollinearity in a multiple regression, we calculated the variance inflation factor (VIF) values for all independent variables. To test whether loneliness scores decreased and intention to cope with loneliness scores increased after an intervention, we constructed two multiple regression models by using “four trial arms” as the categorical independent variable and “loneliness scores after an intervention” as the dependent variable, while controlling for “loneliness scores before an intervention” in one model. In the second model, we used “intention to cope with loneliness scores after an intervention” as the dependent variable, while controlling for “intention to cope with loneliness scores before an intervention”. Following the suggestion by Senn^61^, we used loneliness scores and intention to cope with loneliness scores before an intervention as covariates in both models to adjust for the baseline value. We compared each intervention arm to the control arm by using dummy coding for four trial arms. We also added the covariates including self-esteem, self-efficacy, hope, age, gender, and years of education. To calculate pairwise differences, Bonferroni’s correction was applied. For secondary outcomes’ analyses, we built up four linear models by using “four trial arm” as independent variable (the control arm as a reference) and each secondary outcome as a dependent variable. Lastly, we used independent *t* tests and calculated two-tailed *p* values to compare participants’ emotional responses to the edutainment and written messages.

### Supplementary information

Supplementary Information

## Data Availability

The datasets used during the current study will be available from the corresponding authors on reasonable request.

## Code availability

Code for the statistical analyses will be available from the corresponding authors on reasonable request.
