# Supplementary material for: Evidence-based health messages increase intention to cope with loneliness in Germany: a randomized controlled online trial
Source: NPJ Digit Med. 2024 Apr 29;7:105. doi: 10.1038/s41746-024-01096-7 (PMC11059282; doi:10.1038/s41746-024-01096-7)
Supplement: Supplementary file 1 — Supplementary Information [file 41746_2024_1096_MOESM1_ESM.pdf]

Supplementary Information

Power Analysis

To estimate an effect size, we calculated a repeated measures ANOVA based on our pilot study[31]. We compared the effect of each of the 4 conditions (between-subjects factor), post and prior assessment (within-subjects measures), on loneliness scores. In our pilot study[31], there were no statistically significant difference in loneliness scores between the four conditions ($F(3, 1) = 0.92$, p-value= 0.43, partial eta squared (η^2_p) = 0.01103281.

We utilized the estimated effect size to determine the necessary sample size for our current study. We converted the partial eta squared (η^2_p) value to Cohen's f using the formula: f = sqr (eta^2 / (1 - eta^2)) (Cohen, 1988). This resulted in Cohen's f = 0.1056215. According to Jacob Cohen's guidelines, effect sizes of 0.10, 0.25, and 0.40 represent small, medium, and large effect sizes, respectively. Additionally, we calculated the correlation between pre- and post-scores of loneliness, yielding a correlation coefficient (r) of 0.929808.

The resulting effect size, as well as correlation among repeated measures, was entered into the statistical software G*Power, see below:

F tests - ANOVA: Repeated measures, between factors

Analysis:	A priori: Compute required sample size		
Input:	Effect size f	=	0.1056215
	α err prob	=	0.05
	Power (1-β err prob)	=	0.95
	Number of groups	=	4
	Number of measurements	=	2

	Corr among rep measures	=	0.929808
Output:	Noncentrality parameter λ	=	17.2500111
	Critical F	=	2.6108826
	Numerator *df*	=	3.0000000
	Denominator *df*	=	1488
	Total sample size	=	1492
	Actual power	=	0. 9504153

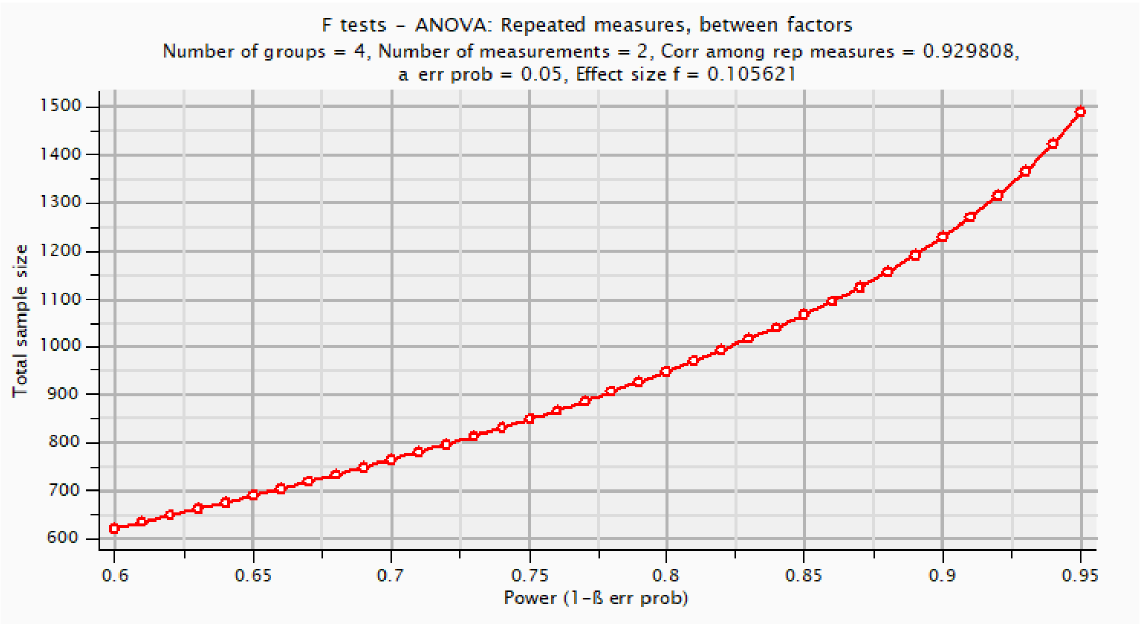

Supplementary Figure 1. Power analysis. The power that can be expected, depending on a relatively small effect size (f = .11), a high correlation among the repeated measures (r = .93) and according to different sample size.

Considering a drop-out rate of 10%, we determined the necessary sample size to be 1641 participants.
